# Directed Evolution of a Homodimeric Laccase from *Cerrena unicolor* BBP6 by Random Mutagenesis and In Vivo Assembly

**DOI:** 10.3390/ijms19102989

**Published:** 2018-09-30

**Authors:** Ji Zhang, Fuying Ma, Xiaoyu Zhang, Anli Geng

**Affiliations:** 1 Key Laboratory of Molecular Biophysics of MOE, College of Life Science and Technology, Huazhong University of Science and Technology, Wuhan 430074, China; zji5@np.edu.sg (J.Z.); mafuying@hust.edu.cn (F.M.); 2 School of Life Sciences and Chemical Technology, Ngee Ann Polytechnic, Singapore 599489, Singapore

**Keywords:** *Cerrena unicolor*, laccase, homodimer, α-factor, low-rate mutation, error-prone PCR, in vivo assembly, enzyme directed evolution, protein modelling

## Abstract

Laccases have great potential for industrial applications due to their green catalytic properties and broad substrate specificities, and various studies have attempted to improve the catalytic performance of these enzymes. Here, to the best of our knowledge, we firstly report the directed evolution of a homodimeric laccase from *Cerrena unicolor* BBP6 fused with α-factor prepro-leader that was engineered through random mutagenesis followed by in vivo assembly in *Saccharomyces cerevisiae*. Three evolved fusion variants selected from ~3500 clones presented 31- to 37-fold increases in total laccase activity, with better thermostability and broader pH profiles. The evolved α-factor prepro-leader enhanced laccase expression levels by up to 2.4-fold. Protein model analysis of these variants reveals that the beneficial mutations have influences on protein pKa shift, subunit interaction, substrate entrance, and C-terminal function.

## 1. Introduction

Laccase is a multicopper oxidase that is capable of oxidizing a wide range of substrates, including phenol and aromatic compounds, using only oxygen from air. Laccases are considered as green catalysts of great biotechnological potential with applications in different industrial processes [1]. In the last decade, a heightened interest in the potential use of laccases as biocatalysts to replace conventional industrial processes in the textile industry, pulp and paper industries, and pharmaceutical industries, has been observed [2,3,4]. These enzymes may potentially be applied in other sectors, such as the food and cosmetic industries and for organic synthesis [5,6,7]. The potential use of laccases for industrial and biotechnological purposes is a booming field of research.

Due to the increasing demands of laccase in various applications, many attempts have been made to engineer laccases with enhanced activity towards adverse substrates and/or increased stability to harsh conditions. Enzyme engineering has been demonstrated as an effective strategy for developing biocatalysts with improved features or alerted functions [8,9]. Rational design is a reliable technique that has commonly been used to identify hotspot residues for mutations that are based on prior structural information [10,11]. Santiago et al. [12] successfully combined a computational approach with experimental validation to rationally design an improved laccase for polyaniline production. The turnover rate *k_cat_* of the engineered variant was improved two-fold after evolution. In another case, the sinapic acid binding pocket of a high redox potential chimeric laccase was delimited by performing iterative saturation mutagenesis (ISM) on six residues. The new variants showed altered pH activity profiles and increased turnover rates for sinapic acid and its methyl ester [13].

However, an improvement in biocatalytic activities was achieved using directed evolution due to long-range allostery in proteins, such that distant mutations should be coupled to the active-site mutations [14]. These reports require us to focus on the entire protein instead of simply focusing on the active sites. Random mutagenesis generates great diversity by error-prone PCR and/or DNA shuffling in the absence of structural or mechanistic information. Mate and coworkers successfully converted a thermostable laccase from a white-rot fungus into a blood tolerant laccase through several rounds of directed evolution [15]. To remove the limitations in lignin degradation in ionic liquids, a laccase (Lcc2) from *Trametes versicolor* was evolved for two generations, and mutants (F265S/A318V) displayed approximately 4.5-fold higher activity than the wild type in the presence of 15% (*v*/*v*) ionic liquid and a 3.5-fold higher activity than the wild type in buffer [16]. Scheiblbrandner et al. [17] reported a method that combined traditional directed evolution, semi-rational design and site-directed mutagenesis. Compared to the wild type, the evolved laccase showed a 2.6-fold improvement in half-life and an up to five-fold increase in specific activities at pH 7.5.

Random mutagenesis has generated many proteins with desired properties, but screening is labor-intensive and time-consuming because evolutionary success is dependent on multiple rounds of evolution [18,19]. To overcome the drawbacks described above, an extensive number of methods have been developed to perform gene diversification over the past two decades, and the most promising strategies combine random and focused mutagenesis, e.g., random mutagenesis followed by site-saturation mutagenesis [8,20]. Random mutagenesis provides a greater chance of accessing functional library members than focused mutagenesis. Additionally, residues that are distant from the active site are also mutation targets due to long-range allostery in proteins. Mutagenic libraries with a high mutation rate have been constructed in previous studies [15,21,22]. However, an individual positive substitution was difficult to be identified as multiple mutations occur in the same variant. Therefore, positive variants with common substitutions have typically been selected as the template for the next generation of mutagenesis until similar positive variants were identified. In addition, saturation mutagenesis was performed based on random mutagenesis, which conferred diversity and increased library quality. However, applying saturation mutagenesis over extensive defined residues still requires the screening of thousands of transformants for sufficient library coverage [17].

Here, in this study, we employed a combinatory approach of random mutagenesis and site-directed mutagenesis for the evolution of a novel homodimeric laccase from *Cerrena unicolor* BBP6, LacA. The native signal sequence of gene *lacA* was replaced with the prepro-leader of α-factor (α*lacA*), and the fusion protein (αLacA) was subjected to two rounds of evolution. A high throughput screening assay that is based on the oxidation of 2,2′-azino-bis(3-ethylbenzothiazoline-6-sulfonic acid) (ABTS) was validated, and the best performing mutants were characterized. Finally, the effects on pH profile, thermostability and kinetics were evaluated in relation to protein structure. To the best of our knowledge, the present study is the first to report the directed evolution of a homodimeric fungal laccase.

## 2. Results and Discussion

### 2.1. Starting Point for Enzyme Evolution

**Figure 1:**
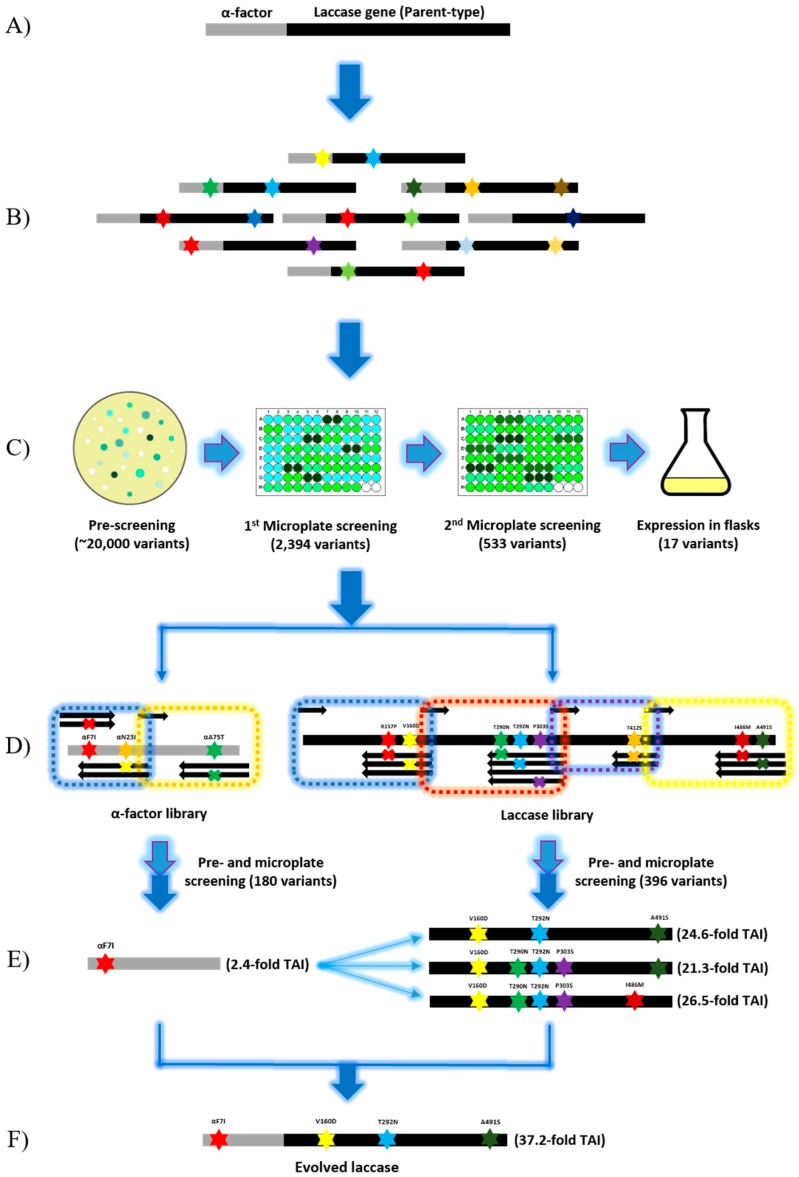
Schematic presentation of laccase directed evolution by random mutagenesis and in vivo assembly in *S. cerevisiae*. (**A**) Generation of the parent-type laccase gene (α*lacA*) by replacing the original laccase signal peptide sequence with α-factor prepro-leader. (**B**) Generation of the mutagenic library of α*lacA* by error-prone PCR using the GeneMorph II Random Mutagenesis kit at a mutation rate of between 0 to 4.5 mutations per 1000 bp. Stars in different colors represent mutations. (**C**) High throughput screening of the mutagenic library of α*lacA*. Light blue arrows represent inoculation. White, light green, and dark green dots on agar plates respectively represent transformants without laccase activity, with low activity and high activity. (**D**) In vivo assembly of α-factor library and laccase library separately in *S. cerevisiae*. Dotted boxes in different colors represent DNA fragments amplified by using different degenerate primers. (**E**) Identification of the best mutation combinations for each library. Light blue fork represents combination of the evolved α-factor and laccase mutants. (**F**) Confirmation of the best variant of α*lacA*.

LacA is a homodimer secreted by a novel white-rot fungus *Cerrena unicolor* BBP6 [23]. LacA is stable at neutral and alkaline pH values. It presents optimal activity at higher temperatures to different substrates than the laccases from other fungal sources [23]. Moreover, native LacA has outstanding affinity to ABTS (*K_m_* = 49.1 µM) when compared to other typical substrates, such as 2,6-dimethoxyphenol (2,6-DMP) (*K_m_* = 3430.8 µM) and guaiacol (*K_m_* = 1238.6 µM) [23]. These properties suggest that LacA is suitable as a starting enzyme of directed evolution, as the promising results of the desired improved function could be achieved without jeopardizing the original function of the enzyme [24]. Additionally, *S. cerevisiae* was chosen as the laccase expression host and ABTS was confirmed as the screening substrate. Because the heterologous expression level of native laccase is undetectable in *S. cerevisiae*, the native signal peptide (NSP) was replaced by prepro-leader of α-factor to enhance laccase expression (Figure 1A) [21]. Laccase activity was increased to approximately 0.33 U L^−1^ by the expression of the fusion enzyme αLacA. Although such laccase activity was too low for enzyme purification, it was detectable and it could be used for laccase variants screening.

In the present study, random mutagenesis with a low mutation rate followed by in vivo assembly was demonstrated. A mutagenic library with a mutation rate of 0–4.5 mutations per 1000 bp was generated by error-prone PCR (epPCR). Thereafter, a few positive variants were selected, and limited mutations were identified as positive on each variant. Finally, all of the positive mutations were re-assembled in *S. cerevisiae* to generate the best combinations. This strategy reduced random mutagenesis to one round and significantly minimized the library size for site-directed mutagenesis. If specific activity towards ABTS can be further improved, then the present strategy will be more reasonable and convincing.

### 2.2. Random Mutagenesis

A random mutagenic library was generated by epPCR while using the GeneMorph II Random Mutagenesis kit (Agilent Technologies, Santa Clara, CA, USA) (Figure 1B). Approximately 20,000 transformants were pre-screened on agar plates for secreted laccase activity. Among the transformants, 2394 variants with green halos were screened in 96-well microplates, and 22.2% of the variants (533 variants) showed higher activity at pH 4.0 compared to that of parent-type αLacA. To confirm the results, such positive variants (533 variants) were further screened under the same conditions. Eventually, 17 variants that were selected from the second batch of microplate screening were inoculated into 100-mL flasks containing 20 mL of expression medium for fermentation (Figure 1C). Enzyme activity assay identified the top eight mutants with total activity improvements (TAI) ranging from 3.3- to 8.2-fold activity as compared with that of the parent-type laccase (Table 1). In total, four mutations (one synonymous mutation) were identified in the α-factor prepro-leader, and thirteen mutations (5 synonymous mutations) were detected in the laccase gene (Table 1). Corresponding to data in the Codon Usage Database (http://www.kazusa.or.jp/codon/), four of the six synonymous mutations favored codon usage in *S. cerevisiae*, which improved the translation and expression of the laccase gene in yeast [25,26].

**Figure 2:**
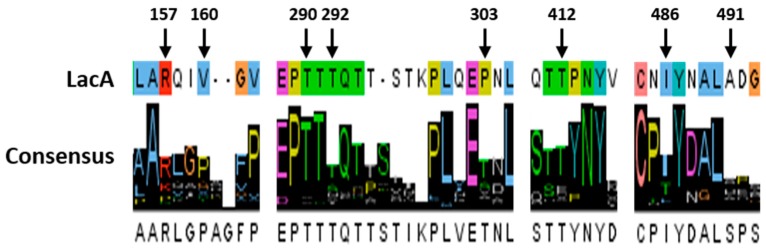
Amino acid consensus results for the alignment of 100 protein sequences with the sequence of LacA. Colors represent different residues generated automatically by the software.

Since *S. cerevisiae* was used as a cell factory rather than an expression host for the final evolved laccase, all of the synonymous mutations would not be integrated into the laccase gene for the next round of evolution despite the fact that these mutations might contribute to activity improvements. As the objective of evolution is performance improvement instead of function alteration, mutations are predicted to occur in non-conserved regions [27,28]. A consensus approach was employed to investigate the mutation locations. The alignment of 100 laccase proteins with the parent laccase was conducted, and the consensus results showed that sites R157, V160, T292, P303, T412, I486, and A491 had no obvious consensus (Figure 2). These sites were therefore considered to be potential sites for further mutagenesis. Additionally, the mutations that were found in α-factor prepro-leaders were extremely relevant because these mutations enhanced laccase secretion and could they serve as universal signal peptides in yeast.

### 2.3. In Vivo Assembly (IVA)

According to the results that are shown in Table 1, only limited mutations (1–3 mutations) were identified in each positive variant. Thereby, three mutations in α-factor and eight mutations in the laccase gene were confirmed as beneficial mutations and selected for site-directed mutagenesis. Assuming that site-directed mutagenesis was applied for these 11 positions, a library containing at least 2000 variants was generated and screened. Therefore, these variants were grouped into two libraries: (A) α-factor library (Fα7, Nα23 and Aα75) and (B) laccase library (R157, V160, T290, T292, P303, T412, I486, and A491). Each position had only two possibilities, the native residue and the substitution obtained from random mutagenesis. We took advantage of the high frequency and efficiency of homologous DNA recombination in *S. cerevisiae* machinery to ligate fragments and assemble mutations [29]. These two libraries, containing 180 and 396 transformants, were screened (Figure 1D). Variants with high activity were rapidly identified through the same screening procedures. The mutation αF7I in α-factor prepro-leader was identified in several variants with TAI ranging from 1.6- to 2.4-fold (Figure 1E). The mutations Nα23I and Aα75T were not found in the top-performing variants. The best variants from library B are listed in the Table 2, showing that IVA189, IVA205, and IVA264 exhibited increases in total activity of 24.6-fold, 21.3-fold, and 26.5-fold, respectively, as compared to the parent-type laccase. These three variants shared mutations V160D and T292N, whereas mutations R157P and I486M were absent. Although only three mutations were found in IVA189, this protein exhibited the higher improvement than IVA205 in activity. The above results indicate that the proper mutation combination is more significant than the number of mutations. The assembly results suggest that beneficial/negative mutations might be buried among several mutations and that additive effects might not occur between the positive mutations. Therefore, to obtain the best combination of mutations, a general directed evolution requires a few generations to evolve new positive mutations and screen out negative mutations. However, the present strategy solves this problem by simple mutation assembly.

### 2.4. Characterization of Laccase Variants

Throughout the experiments, the protein content in the crude enzyme that was generated by *S. cerevisiae* was almost constant, 15.1 ± 1.5 µg/mL, for the parent-type laccase and its variants. However, the laccase activity varied from ~0.3 to ~12 U L^−1^. Because of such low protein content, crude enzymes were used for laccase characterization and kinetic studies. The pH profiles, thermostability, and kinetics parameters of 13 selected variants were investigated.

#### 2.4.1. The pH Profile of Evolved Laccases

**Figure 3:**
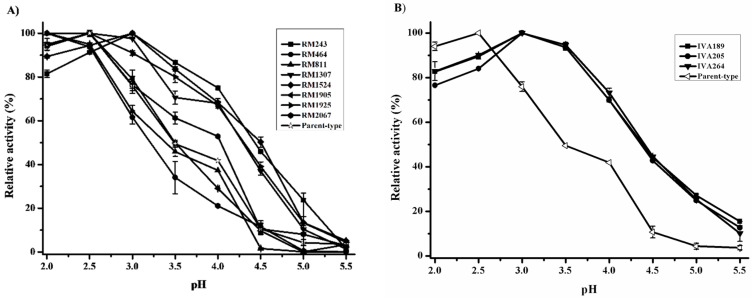

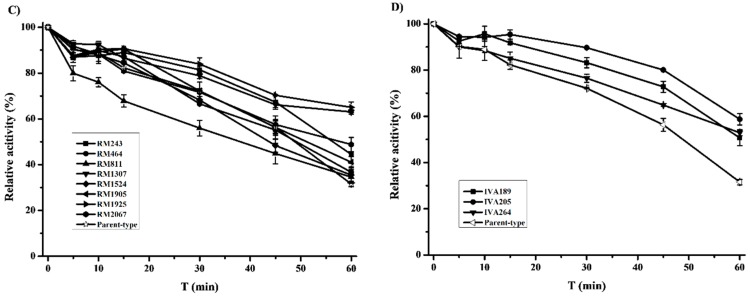
Effect of the evolved laccases on pH profile and thermostability. (**A**) pH profile of the evolved laccases from the random mutagenic library. (**B**) pH profile of the evolved laccases from the laccase library after in vivo assembly. (**C**) Thermostability of the evolved laccases from the random mutagenic library. (**D**) Thermostability of the evolved laccases from the laccase library after in vivo assembly. The results represent the mean ± standard deviation of duplicate independent experiments (*p* < 0.01).

LacA has poor stability at pH values below 3.0 [20]. The laccase activity assay screenings were therefore performed at pH 4.0, which is an appropriate pH value for the present laccase applications. Therefore, a consequence of the selective pressure applied in the evolution should shift the pH profile to pH 4.0. The optimal pH of the parent laccase is 2.5, which was the same as that of the native laccase from *Cerrena unicolor* BBP6, and 41.8% relative activity was detected at pH 4.0 (Figure 3A). The pH profile of the mutants RM243 and RM1524 exhibited a switch in optimal pH from 2.5 to 3.0, retaining at least 75% and 68% relative activity between pH 2.0 and 4.0, respectively. The mutants RM1307, RM1905, and RM1925 showed the same optimal pH at 2.5, whereas the mutants RM1307 and RM1925 presented broader pH profiles and retained almost 40% residue activity at pH 4.5. Regardless of the improved activity, the mutants RM464, RM811, and RM2067 had narrow pH profiles and they showed almost no residual activity above pH 4.5. The optimal pH of these mutants was shifted to pH 2.0 (Figure 3A). Expectedly, the pH profile of the three positive mutants from the in vivo assembly libraries was homogeneous. The mutants presented the same optimal pH at 3.0, and 10–15% activity remained at pH 5.5 (Figure 3B). Accordingly, the beneficial mutations, V160D and T292N, were found in these mutants after assembly.

#### 2.4.2. Thermostability

The thermostability was conducted by incubating the evolved laccases at 50 °C for 60 min. In contrast to the pH profiles, the thermostability of most mutants from the random mutagenic library was 100% conserved. The results showed that all mutants retained over 30% activity on ABTS upon incubation at 50 °C for 1 h. Notably, the mutants RM1307 and RM1925 displayed increased stability from 10–60 min, and the residual activity was more than two-fold that of the parent type. In contrast, the decreased stability of RM811 was found between 10–50 min (Figure 3C). As shown in Figure 3D, the thermostability of the evolved laccases was further improved after reassembling the above mutations. The relative activity of the evolved laccases was retained from 51% to 67% after a 1-h incubation at 50 °C. Moreover, the negative mutation R157P was absent in the mutated laccases after assembly.

#### 2.4.3. Kinetics of Evolved Laccases

The kinetics parameters of the evolved laccases were investigated at pH 4.0 using ABTS as the substrate. Unfortunately, we were not able to obtain purified laccases due to the low expression level of laccase (parent-type laccase: ~0.33 U L^−1^) in *S. cerevisiae*. To this end, the supernatant of the positive variants obtained from two-round evolution was kinetically characterized. All of the kinetics parameters and specific activities of parent-type and mutated laccases to ABTS are listed in Table 1 and Table 2. The substrate binding constant (*K_m_*), turnover rate (*k_cat_*), and catalytic efficiency (*k_cat_*/*K_m_*) were 205.7 µM, 2208.2 s^−1^ and 10.9 µM^−1^ s^−1^, respectively for the recombinant αLacA expressed in *S. cerevisiae*. Regardless of the mutant libraries, the *K_m_* values of the most evolved laccases were enhanced to 60–90 µM, except for the mutants RM811, RM1905, and RM2067, which displayed *K_m_* values over 140 µM. Therefore, R157P and I486M were screened out after assembly. The mutants RM243 and RM1524 displayed 3.9- and 3.5-fold increases in *k_cat_* and 11.9- and 12.3-fold increases in *k_cat_*/*K_m_*, respectively, when compared to those of the parent type. As shown in Table 2, the mutations V160D and T292N expectedly occurred in all the selected laccase mutants after in vivo assembly. The mutants IVA189, IVA205 and IVA264 exhibited outstanding kinetics parameters compared to those of other evolved laccases and the parent type. The substrate affinity constant (*K_m_*) was 69.8, 85.3 and 64.0 µM, respectively. The turnover rates (*k_cat_*) of such variants were 9.3-, 9.4-, and 7.3-fold greater, respectively, than that of the parent-type laccase. Accordingly, the *k_cat_*/*K_m_* values were increased up to 27.0-fold. The above results suggest that the performance of the mutants is not proportional to the number of substitutions. Interestingly, the kinetics performance of the laccase variants towards ABTS was considerably enhanced, while their affinity constants were similar to those of other fungal laccases [17,30].

In conclusion, we report a two-step mutagenesis approach to obtain engineered laccase variants with increased total activity and catalytic efficiency towards the substrate ABTS while retaining good thermostability and broader pH profiles when compared to those of the parent-type enzyme.

### 2.5. Effect of α-Factor Prepro-Leader in Laccase Production

**Figure 4:**
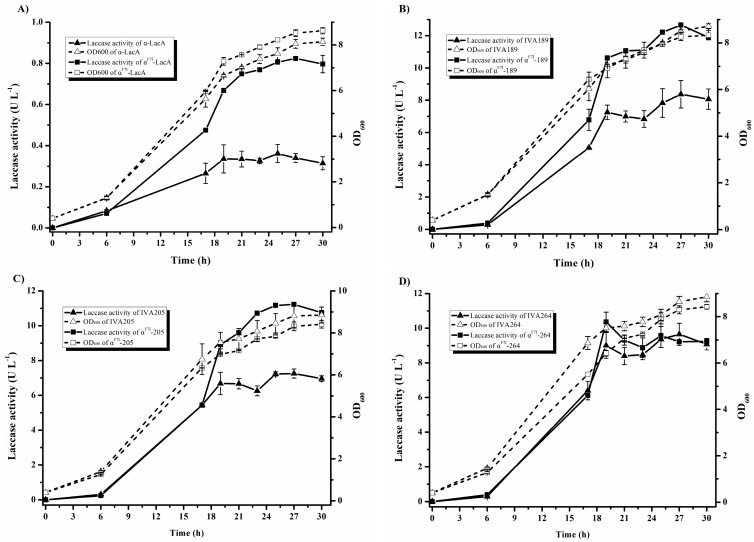
Cell growth and laccase production in *S. cerevisiae* BJ5465 expressing the parent-type laccase, LacA, and its mutants with the native α-factor and the evolved α-factor. (**A**) The parent-type laccase, LacA. (**B**) Mutant IVA189. (**C**) Mutant IVA205. (**D**) Mutant IVA264. The results represent the mean ± standard deviation of duplicate independent experiments (*p* < 0.01).

The α-factor prepro-leader is frequently employed in the heterologous expression of laccases in *S. cerevisiae* [15,22,31]. In the present study, the evolved *S. cerevisiae* α-factor prepro-leader (αF7I) from the α-factor library was tested to investigate its effect on the secretion of the evolved laccase mutants. The best three evolved variants (IVA189, IVA205, and IVA264) were fused to the evolved α-factor. The fusion genes were generated by in vivo ligation and named α^F7I^-189 (αF7I/V160D/T292N/A491S), α^F7I^-205 (αF7I/V160D/T290N/T292N/P303-S/A491S), and α^F7I^-264 (αF7I/V160D/T290N/T292N/P303S/T412S). As shown in Figure 4A, the evolved α-factor αF7I improved the laccase activity to 0.82 U L^−1^ after 27 h of cultivation, which was 2.4-fold that of native αLacA. Similar results were obtained with α^F7I^-189 and α^F7I^-205, showing approximate 1.5-fold total activities while using the evolved α-factor. Compared with that of the parent-type αLacA, the TAI were improved 33-fold and 37-fold, respectively (Figure 4B,C). Additionally, the laccase activity of α^F7I^-264 was close to that obtained with native α-factor, with only 15% improvement being achieved at 19 h (Figure 4D). Notably, the activity improvement in the laccase mutants by the evolved α-factor was lower than that in native laccase (2.4-fold) because the mutations in laccase also contributed to laccase secretion [22,32]. Mutation αF7I was located in the hydrophobic domain of the pre-leader region, consistent with the reported mutations [15,22]. The pre-leader is involved in directing the nascent secretory protein to the endoplasmic reticulum (ER) membrane [33]. The correlation between the evolved α-factor and the laccase mutants indicates that the evolved α-factor αF7I promotes the secretion of laccase.

### 2.6. Structure Analysis of the Evolved Laccase

**Figure 5:**
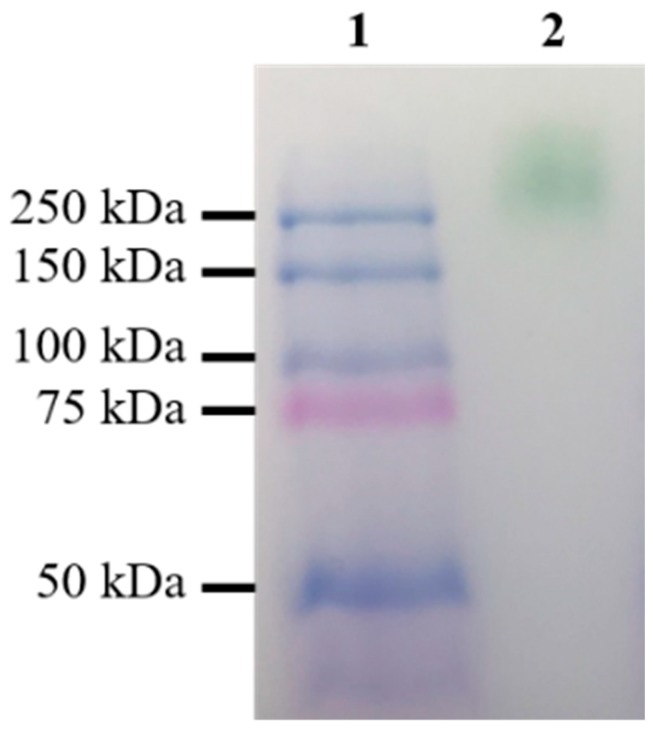
Native-PAGE of the parent-type laccase produced by *S. cerevisiae* BJ5465. Lane 1, Precision Plus Protein Dual Color S tandards; lane 2, the parent-type LacA.

**Figure 6:**
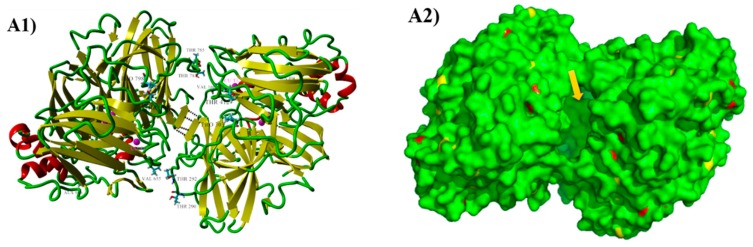

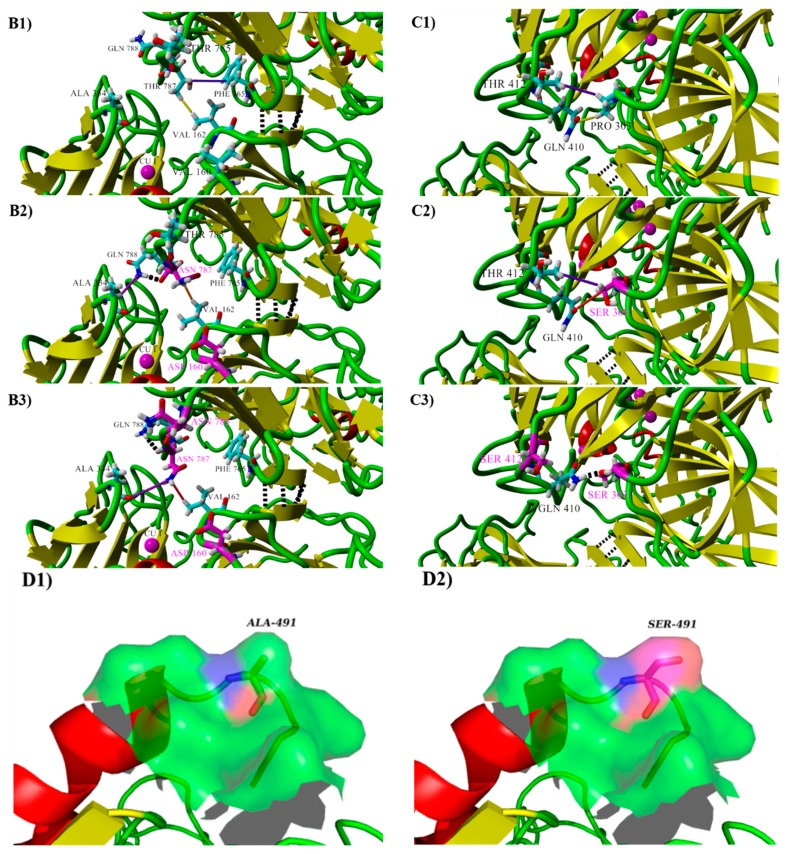
Location of different residues in the parent laccase and the mutants introduced by directed evolution. Protein model of the parent laccase based on homodimeric laccase 3X1B (**A1**). Solvent accessible surface of the LacA homodimer. The yellow arrow points at the substrate entrance. (**A2**). Details of the mutation complex (in magenta) in variants (**B2**,**B3**,**C2**,**C3**,**D2**) compared with the corresponding residues in the native laccase (**B1**,**C1**,**D1**). Magenta spheres represent Cu atoms. The vdW interactions are shown as yellow, blue, red, and purple sticks. H-bonds are shown as black dashes.

The parental laccase from *Cerrena unicolor* BBP6 was identified as a homodimer with a molecular mass of ~110 kDa in zymograph in our previous study [20]. The result of native polyacrylamide gel electrophoresis (PAGE) showed that the size of recombinant laccase was over 250 kDa (Figure 5). The high molecular mass suggests that this enzyme was expressed with a high degree of glycosylation in *S. cerevisiae*, as is consistent with the results of a previous report [22]. It also indicates that the recombinant laccase is still a homodimer. The LacA homodimer has two subunits, subunits A and B, corresponding to protein sequence chains A and B. The homodimeric laccase (Protein Data Bank ID: 3X1B) sharing 68.7% identity to LacA was used as the template for protein modeling. The three-dimensional (3D) structures were further repaired with FoldX to correct bad torsion angles and van der Waals clashes and to minimize the total energy. The final structure of the homodimer is shown in Figure 6(A1), demonstrating that the connection of subunits A and B was primarily mediated by four hydrogen bonds between the β-sheets at positions 266–268 and 761–763.

After repairing the protein modeling structures, the free energy of folding for the parent laccase (ΔG) and the relative Gibbs free energies of folding between the variants and the parent laccase (ΔΔG) were calculated by using FoldX, and the results are listed in Table 3. The free energy of folding represented the difference in free energy between the folded and the unfolded states [34]. The ΔΔG values listed in Table 3 reveals that the mutants IVA189, IVA205, and IVA264 had lower free energies of folding and lower interaction energies of subunits. This observation was consistent with the thermostability results (Figure 3D) and the protein stability prediction by FoldX [35,36]. Moreover, the most significant relative energy change between the parent-type and the mutants was the energy of inter-residue van der Waals clashes (ΔΔG = −46.35 to −39.64 kcal mol^−1^) and the energy of intra-residue van der Waals torsional clashes (ΔΔG = −20.18 to −17.36 kcal mol^−1^). The energy term of van der Waals clashes may represent physically relevant unfavorable interactions, as well as errors that are inherent to the geometry of the homology model [35]. The lower this value is, the more reasonable and stable the protein structure.

In addition to ΔG, the pKa value is also critical to the pH dependence of enzyme [37]. In the present study, pKa values were calculated by a web server DelPhiPKa using default parameters at pH 4.0. As shown in Table 4, the pKa values of IVA189, IVA205, and IVA264 were increased 9.87, 11.82, and 12.30, respectively. These changes may cause approximately 0.5 units of optimal pH-shift for the evolved laccases.

The homodimeric structure of LacA indicates any mutations in the gene will result in substitutions on both subunits. As shown in Figure 6(A1), the mutations V160D, T290N, T292N, P303S, T412S, and A491S on subunit A were also reflected on subunit B as V655D, T785N, T787N, P798S, T907S and A986S. V160D (V655D) occurred in all positive mutants and it was located at the surface coil of the protein, approximately 11 Å from the type I copper (Cu I) site. The mutation of this position changed a Val residue to Asp, which belongs to the titratable group. Hence, V160D and V655D significantly increased the pKa value by over eight units, and the total difference in the protein pKa values was primarily contributed by this exchange (Table 4). This mutation changed the pH profile and improved the catalytic performance of laccase at pH 4.0, as demonstrated by the decrease in *K_m_* (RM1524).

T785N and T787N (T290N and T292N) were located at the interface of the two subunits and at the sides of substrate entrance (Figure 6(A1,A2)). The van der Waals (vdW) interactions are considered as one of the main contributors to the stabilization of protein dimers. The vdW potentials of atoms display a shallow attractive energy minimum at radii of 1.3–2.2 Å, and a strong repulsion at shorter distances [38]. Therefore, the vdW interactions between residues was generated by YASARA (version 17.12.24) at vdW radii less than 2.2 Å. Thr787 is the key residue; it showed vdW contacts with Phe765 in the same subunit B and with Val162 being located in subunit A (Figure 6(B1)). The exchange of Asn787 retained vdW interaction with Val162, but it rendered a new hydrogen bond (H-bond) with Gln788. The new H-bond shifted the direction of the Gln788 side chain and resulted in new vdW contact with Ala334 located in a coil in subunit A (Figure 6(B2)). The mutation T785N further modified the geometry of this region. The H-bond between T787N and Gln788 was retained, whereas the vdW interaction with Ala334 no longer existed. However, a new vdW contact was generated between Asn787 and Ala334 (Figure 6(B3)). The above protein model analysis suggests that mutations T785N and T787N improved the vdW interactions between subunits A and B, which play a key role in the stability of the homodimer.

Pro303 (798) is located in a turn next to the entrance of the substrate channel, and Thr412 (907) is located in a neighboring coil in the same subunit (Figure 6(C1)). It is highly unlikely that the mutation P303S would enhance the interaction of this region alone (Figure 6(C2)), as is reflected by the slight decrease in the *K_m_* of mutant RM2067 (αF7I/P303S) (Table 1). Further improvement was observed when Thr was replaced by Ser at position 412. The mutant T412S donated space to Thr410 by shortening its side chain. Apart from a new H-bond formed between Gln410 and Ser303, the notable implication of this mutation was the expansion of the substrate entrance on both sides (Figure 6(C3)). Thus, the mutations P303S and T412S provided a more stable and expanded entrance for substrates, leading to a decrease in the *K_m_* and an increase in the *k_cat_* as compared to those of the parent type.

Ala491 (986) is located in the C-terminal coil at the protein surface (Figure 6(D1)). The mutation on (A491S) this tail has previously been confirmed to affect the functions of laccases (Figure 6(D2)). The protein structure of a laccase POXA1b from the ascomycete *Melanocarpus albomyces* revealed that the extension of the C-terminus opens the solvent channel for the entrance of oxygen molecules and the subsequent exit of water molecules [39]. Additionally, Autore et al. [40] reported that the C-terminal tail of POXA1b might affect the enzyme stability properties.

All of the beneficial mutations investigated in the present study were mapped far from the binding pocket. It is highly improbable that such mutations could be predicted by rational design. However, random mutagenesis determined the functional relevance of these previously unknown residue positions in laccase [22]. Notably, LacA is a homodimer, and this structure likely undergoes pH-dependent dimerization [41]. Some studies have revealed correlations between stability and enzymatic catalysis, although no direct evidence affecting substrate binding was shown in the crystal structure [42]. However, this correlation makes sense, as the more stable the protein, the better it works.

### 2.7. Comparison of Laccases Evolution

In recent years, the strong potential of laccase application in green industry has motivated research on laccase engineering (Table S1). The present study is the first report on the directed evolution of a homodimeric laccase. Through random mutagenesis with low mutation rates followed by in vivo assembly, variants with improved kinetics constants and specific activities were successfully obtained by screening a library of ~3500 clones, which was smaller than that reported in most previous studies (Table S1). The fold-changes in the kinetics parameters were significantly better than those reported with two or less rounds of evolution (Table S1). The specific laccase activity was also improved by up to 29-fold, although the total laccase activity was improved only 37-fold, which was much lower than the findings reported in some previous studies (Table S1). Nevertheless, such an improvement was significant for two-round enzyme-directed evolution. More rounds of evolution and additional improvements in protein secretion/production might further enhance the laccase titer. The results listed in Table S1 suggest that the laccase evolution strategy demonstrated in the present study is effective and quite comparable to those that are described in previous reports.

## 3. Materials and Methods

### 3.1. Vector, Strains and Media

A novel *Cerrena unicolor* BBP6 laccase gene *lacA* (NCBI GenBank Accession No. KY400275.1) was amplified by using Marathon cDNA Amplification Kit (Clontech, CA, USA). Gene *lacA* fused with prepro-leader of α-factor (α*lacA*, 1743 bp) and an ampicillin resistance shuttle vector pYES2-α*lacA* under the control of *GAL1* promoter was obtained from a previous experiment in the lab. Protease-deficient *S. cerevisiae* BJ5465, ATCC 208289 (American Type Culture Collection, Rockville, MD, USA) was used as the host for laccase heterologous expression. *Escherichia coli* DH5α (Invitrogen, Carlsbad, CA, USA) cells were used for plasmid amplification.

The screening medium that was used for selective plates (1 L) contained 50 mL 13.4% (*w*/*v*) sterile yeast nitrogen base, 1.92 g yeast synthetic dropout medium supplement without uracil, 20 g Bacto agar, 100 mL 20% (*w*/*v*) sterile raffinose, 100 mL 20% (*w*/*v*) sterile galactose, 1 M sorbitol, 0.2 mM CuSO_4_, 0.4 mM ABTS and double-distilled H_2_O (ddH_2_O). The growth medium (1 L) contained 50 mL 13.4% (*w*/*v*) yeast nitrogen base, 1.92 g of yeast synthetic dropout medium supplement without uracil, 100 mL sterile 20% (*w*/*v*) raffinose, and 850 mL ddH_2_O. The expression medium had the same composition as the screening medium without Bacto agar, sorbitol and ABTS. All the chemicals were of the analytical grade and they were obtained from Sigma-Aldrich (Singapore) unless otherwise stated.

### 3.2. Random Mutagenesis

A mutagenic library (~20,000 mutants) was generated with a GeneMorph II Random Mutagenesis kit (Agilent Technologies, Santa Clara, CA, USA) using α*lacA* as the template. The library was constructed at a mutation rate of between 0 to 4.5 mutations per 1000 bp. Error-prone PCR (epPCR) was performed in a T100 thermocycler (Bio-Rad Laboratories, Woodinville, WA, USA) at a final volume of 50 µL containing 500 nM each primer, 814 ng template, dNTPs (0.2 mM each), and 2.5 units of Mutazyme II DNA polymerase. PCR was performed under the following conditions: 95 °C for 2 min (1 cycle); 95 °C for 0.5 min, 50 °C for 0.5 min, and 72 °C for 2 min (30 cycles); and, 72 °C for 10 min (1 cycle). The primers OL_pYEα-F and OL_lac-R (Table S2) used for amplification had overhangs of 44 and 66 bp homologous to the vector pYES2 to enable in vivo ligation. PCR products and the linearized pYES2 (linearized by *EcoRI* and *XbaI*) were purified and concentrated after electrophoresis by using a gel extraction kit (Omega Bio-tek, Norcross, GA, USA). Subsequently, 300 ng of PCR fragments were mixed with the linearized vector (100 ng) and transformed into *S. cerevisiae* BJ5465 competent cells by using the Gene Pulser Xcell System (Bio-Rad Laboratories, Woodinville, WA, USA). All of the transformed cells were spread onto selection plates and incubated for three days at 30 °C.

### 3.3. In Vivo Assembly

Libraries were constructed by in vivo assembly in *S. cerevisiae* taking advantage of its efficient DNA in vivo homologous recombination capacity [29]. Primers were designed based on the amino acid substitutions in the positive variants that were selected from the last generation. In total, 11 residues were involved and divided into two groups, α-factor library and laccase library. All the primers used in this section are listed in Table S2.

#### 3.3.1. α-Factor Library

The short fragments αF1 was generated by modified PCR using degenerate primers αF_1F and αF_1R. PCR was conducted in a final volume of 20 µL containing 5 µM each primer, dNTPs (0.2 mM each), and five units of Bestaq DNA polymerase (Applied Biological Materials (ABM) Inc., Vancouver, BC, Canada). The PCR conditions for αF1 were 94 °C for 3 min (1 cycle); 60 °C for 0.5 min; and, 72 °C for 0.5 min (1 cycle); 72 °C for 5 min (1 cycle). The 20 µL of reaction mixture was directly used for transformation. The fragment αF2 was amplified using degenerate primers αF_2F and αF_2R (Table S2). The 50 µL reaction mixtures contained 500 nM each primer, 100 ng template, dNTPs (0.2 mM each) and 5 units of Bestaq DNA polymerase. The PCR conditions for αF2 amplification were 94 °C for 3 min (1 cycle); 94 °C for 10 s, 60 °C for 0.5 min, and 72 °C for 0.5 min (30 cycles); and, 72 °C for 5 min (1 cycle). PCR fragments αF2 were analyzed by electrophoresis on agarose gels and they purified by using a gel extraction kit (Omega Bio-tek, Norcross, GA, USA). The backbone of pYES2-*lacA* (without α-factor) for in vivo ligation was created by digesting vector pYES2-α*lacA* with *EcoRI* and *XhoI* (Thermo Fisher Scientific, Waltham, MA, USA) at 37 °C for 3 h. The fragments αF2 and pYES2-*lacA* were further analyzed on an agarose gel by electrophoresis and purified by using a gel extraction kit (Omega Bio-tek, Norcross, GA, USA). The mixture of αF1 (17 ng), αF2 (30 ng), and pYES2-*lacA* (100 ng) was transformed into *S. cerevisiae* BJ5465 competent cells by using the Gene Pulser Xcell System. All of the transformed cells were spread onto selective plates and incubated for three days at 30 °C.

#### 3.3.2. Laccase Library

Amplification of short *lacA* fragments (*lac1*, *lac2*, *lac3* and *lac4*) was achieved using the same procedure of αF2 amplification, as previously described. The primers used are listed in Table S2. The backbone of pYES2-αfactor (without laccase gene) for in vivo ligation was created by digesting vector pYES2-α*lacA* with *XhoI* and *XbaI* (Thermo Fisher Scientific, Waltham, MA, USA) at 37 °C for 3 h. The mixture of *lac1* (50 ng), *lac2* (40 ng), *lac3* (30 ng), *lac4* (25 ng), and pYES2-αfactor (100 ng) was transformed into *S. cerevisiae* BJ5465 competent cells using the Gene Pulser Xcell System. All of the transformed cells were spread onto the selective plates and incubated at 30 °C for three days.

### 3.4. High Throughput (HTP) Screening

HTP screening was performed according to previous reports, with some modifications [24].

#### 3.4.1. Pre-Screening on ABTS Plates

Positive *S. cerevisiae* transformants were selected on selection plates containing ABTS. Colonies showing green halos after three-day incubation at 30 °C were sub-cultured on 96-well microplates (Greiner Bio-One International, Kremsmuenster, Austria) for high-throughput screening.

#### 3.4.2. Microplate Screening

Colonies selected from pre-screening were transferred with sterile crystal tips to a 96-well microplate containing 50 μL of growth medium in each well. After 1–2 days of incubation at 28 °C and 700 rpm in a thermo-shaker (MRC Ltd., Holon, Israel), 150 μL of the expression medium was added, and the plates were incubated for another 2–3 days. Each variant was grown in two wells; wells H9–H10 were inoculated with the parent type, and wells H11-H12 were not inoculated. The plates were sealed with Parafilm to prevent evaporation. To determine laccase activity in each well, the plates were centrifuged at 4500× *g* and room temperature for 3 min, and 20 μL aliquots of the supernatant were transferred to new plates. A mixture of one millimolar ABTS in 100 mM acetate buffer (pH 4.0) was brought to a final volume of 200 μL, and the absorbance was measured at 420 nm (ɛ420 = 36,000 M^−1^ cm^−1^) in kinetics mode in a microplate reader (Infinite 200 PRO, Tecan Group AG, Männedorf, Switzerland). To confirm the results, positive variants having higher laccase activity than the parent type were selected for the second batch of screening. Each positive variant was grown in three wells, wells H7–H9 were inoculated with the parent laccase, and wells H10–H12 were not inoculated. The plates were assessed by using the same cultivation and assay protocols described above.

#### 3.4.3. Flask Screening

The best variants selected from the second batch of microplate screening were inoculated to a 50 mL centrifuge tube containing 5 mL of the growth medium and were incubated at 30 °C and 200 rpm for two days. An aliquot of the cells was transferred to 20 mL expression medium in a 100 mL flask to an OD_600_ of 0.4. The flasks were incubated at 28 °C and 200 rpm for 1–2 days. Crude broth was harvested, and the cells were precipitated by centrifugation at 4500× *g* for 5 min. The supernatant was used for enzyme activity assays, characterization, and plasmid extraction.

### 3.5. Laccase Activity Assay and Protein Electrophoresis

The activity of laccase was determined according to the previously published methods, with slight modifications [24]. A mixture of 20 µL of crude enzyme and 180 µL of 1 mM ABTS dissolved in 100 mM acetate buffer (pH 4.0) was used to determine the laccase activity. The oxidation of ABTS was spectrophotometrically monitored in the microplate reader (Infinite 200 PRO, Tecan Group AG, Männedorf, Switzerland) by measuring the increase in absorbance at 420 nm (ɛ420 = 36,000 M^−1^ cm^−1^) at room temperature. One unit of enzyme activity (U) was defined as the amount of enzyme required to produce 1 µmol oxidized substrate per min.

Polyacrylamide gel electrophoresis under non-denaturing conditions (native-PAGE) was conducted with a 10% Mini-PROTEAN TGX precast gel (Bio-Rad Laboratories, Hercules, CA, USA) by using the Precision Plus Protein Dual Color Standards (Bio-Rad Laboratories, Woodinville, WA, USA) in a Mini Protein Tetra System (Bio-Rad Laboratories, Woodinville, WA, USA) according to the manufacturer’s instructions. The laccase band was visualized by incubating the gel in 1 mM ABTS and 100 mM sodium acetate buffer (pH 4.0) at room temperature for 20 min.

### 3.6. Characterization of Crude Laccase after Evolution

The optimal pH for laccase activity was evaluated by the varying pH values in 100 mM citrate buffer (pH 2.0–3.0) and 100 mM acetate buffer (pH 3.5–5.5) with ABTS as the substrates. The supernatants (30 µL) of the best variants mixed with 180 µL of 100 mM acetate buffer (pH 4.0) containing 1 mM ABTS were used to determine the laccase activity. Oxidation of ABTS was spectrophotometrically monitored at 420 nm at room temperature in the microplate reader.

The thermostability of the best variants were investigated by incubating supernatants for 0, 5, 10, 15, 30, 45, and 60 min at 50 °C, followed by chilling samples at 4 °C for 10 min and incubating them at room temperature for an additional 5 min. Subsequently, the residual laccase activity was measured, as described above.

The substrate specificities of the variants were estimated by measuring the absorption change at 420 nm in a final volume of 210 µL containing 30 µL supernatant, 100 mM acetate buffer, and 10–1000 µM ABTS at pH 4.0 and 30 °C. The kinetics parameters of the Michaelis-Menten equation were calculated according to Lineweaver-Burk plots. All the assays were conducted in duplicate wells on one 96-well plate.

### 3.7. Plasmid Extraction and DNA Sequencing

Plasmids with the evolved α*lacA* were extracted (Yeast Plasmid Mini Kit, Omega Bio-tek, Norcross, GA, USA) and transferred into *E. coli* DH5α cells. The *E. coli* transformants were spread onto Luria-Bertani (LB) agar plates with 200 µg L^−1^ ampicillin as the selective marker. Subsequently, single colonies were selected and used to inoculate 5 mL LB ampicillin medium overnight at 37 °C and 200 rpm. Subsequently, the plasmids were extracted (NucleoSpin Plasmid EasyPure; Macherey-Nagel GmbH & Co. KG, Düren, Germany). Pure plasmids were sequenced by using a BigDye Terminator (version 3.1) cycle sequencing kit (Thermo Fisher Scientific, Waltham, MA, USA) and were performed at a DNA Sequencing Service (1st BASE, Singapore). The primers used for sequencing were designed by using Primer 5 software (http://www.premierbiosoft.com/primerdesign/) and are listed in Table S2.

### 3.8. Laccase and Laccase Variants Expression in S. Cerevisiae

The evolved prepro-leader of α-factor and the evolved laccases were amplified using the primers αF54_F/R and Lac_uni_F/R, respectively. The linearized plasmid backbone was generated by double digestion using *EcoRI* and *XbaI*. Fragment α-factor (20 ng), evolved laccases (100 ng), and pYES2 backbone (100 ng) were mixed and transformed into *S. cerevisiae* BJ5465. The PCR conditions, electroporation methodology, transformants selection, and inoculation were previously described. Flasks were incubated at 28 °C and 200 rpm for 30 h. Aliquots of the culture supernatant were sampled at different time intervals to measure the enzyme activity.

### 3.9. Sequences Alignment and Protein Modeling

Alignment of 100 laccase proteins that were obtained from NCBI (https://www.ncbi.nlm.nih.gov/) with the parent laccase was conducted using ClustalW (http://www.clustal.org). The 3D structure of the evolved and parent-type laccase LacA were generated by homology modeling using DeepView Project Mode on SWISS-MODEL web server (http://swissmodel.expasy.org/). The template laccase with 68.7% identity to LacA was obtained from *Lentinus* sp. at 1.8-Å resolution (PDB ID: 3X1B) according to similarity alignment of the proteins.

### 3.10. FoldX and pKa Calculation

The 3D models of the parent-type and evolved laccase were analyzed with YASARA viewer (http://www.yasara.org/) by using FoldX (version 4.0) [43] as a plugin tool. Protein models were repaired by a subroutine ‘Repair’ to achieve the minimum energy. Thereafter, the Gibbs free energy (ΔG) of each repaired model was calculated by the subroutine ‘Stability’. The relative free energy of folding (ΔΔG) for the parent-type laccase and mutants was calculated by using an equation that is defined as ΔΔG = ΔG_M_ − ΔG_PT_. Here, ΔG_M_ and ΔG_PT_ are the free energies of folding for the mutant and the parent-type proteins, respectively [35].

Calculation of pKa values was performed with the DelPhiPKa web server, in which a Poisson-Boltzmann based approach was used to calculate the pKa values [44,45]. The pKa shift (ΔpKa) was calculated by an equation defined as ΔpKa = pKa^M^ − pKa^PT^. Here, pKa^M^ and pKa^PT^ are, respectively, the pKa values for the mutant and parent-type proteins.

The solvent accessible surface of the protein structure is shown by PyMOL viewer using repaired protein models.

## 4. Conclusions

Laccases are being increasingly evaluated for use in a variety of biotechnological applications due to their green catalytic properties and broad substrate specificities [22,41]. In the present study, a two-step evolution strategy involving random mutagenesis followed by in vivo assembly was demonstrated. Potential mutations that were found by random mutagenesis at a low mutation rate were directly reassembled in small groups in *S. cerevisiae*. This approach reduced the workload and thereby the screening time. Positive variants with up to 37-fold TAI at pH 4.0 with better thermostability and broader pH profiles were obtained. Furthermore, the fusion of α-factor prepro-leader and mature laccase made laccase expression levels detectable [22]. The protein model analysis of these variants suggests that beneficial mutations have influences on protein pKa shift, subunits interaction, substrate entrance, and C-terminal function. To the best of our knowledge, the present study is the first report on the evolution of a homodimeric fungal laccase. These experiments led to not only more efficient laccase mutants, but also an increased knowledge on the structure-function relationships of a homodimeric laccase. In conclusion, the evolution strategy developed in the present study is effective and it shows potential in the improvement of other enzymes.

## Figures and Tables

**Table 1:** Comparison of the kinetics parameters of evolved laccases from the random mutagenic library at pH 4.0.

Variants	Mutations		Specific Activity (U mg^−1^)	*K_m_* (µM)	*k_cat_* (s^−1^)	*k_cat_*/*K_m_* (µM^−1^ s^−1^)	TAI (Fold)
	Prepro-Leader of α-Factor	Laccase					
Parent-type	-		19.1 ± 1.1	205.7 ± 11.6	2208.2 ± 365.3	10.9 ± 2.4	1
RM243	-	Y(TAC)209Y(TAT), T292N	141.4 ± 9.1	64.8 ± 1.4	8669.5 ± 832.0	134.0 ± 15.7	6.9 ± 0.0
RM464	-	A(GCT)362A(GCC), A(GCG)386A(GCA), A491S	155.3 ± 3.8	90.2 ± 3.4	3787.1 ± 510.9	41.8 ± 4.1	8.2 ± 0.3
RM811	-	Y(TAC)152Y(TAT), R157P	67.3 ± 4.5	167.5 ± 0.0	4596.9 ± 0.0	27.4 ± 0.0	3.3 ± 0.3
RM1307	P(CCG)34P(CCT), αN23I, αA75T	V(GTC)187V(GTA), T290N	77.1 ± 17.3	67.5 ± 1.7	6082.3 ± 619.1	90.5 ± 11.5	3.9 ± 1.1
RM1524	-	V160D	129.2 ± 11.2	59.8 ± 2.2	7658.0 ± 1418.4	129.2 ± 28.4	5.2 ± 0.7
RM1905	-	I486M	84.2 ± 4.9	146.9 ± 14.6	4962.2 ± 699.5	33.6 ± 1.4	4.1 ± 0.2
RM1925	-	T412S	119.1 ± 4.9	79.8 ± 12.7	6260.4 ± 442.3	81.5 ± 18.5	6.1 ± 0.4
RM2067	αF7I	P303S	119.2 ± 18.5	143.0 ± 0.0	3865.8 ± 0.0	27.0 ± 0.0	5.6 ± 0.3

The results represent the mean ± standard deviation of duplicate independent experiments.

**Table 2:** Kinetics constants of evolved laccases from the laccase library after in vivo assembly at pH 4.0.

Variants	Mutations	Specific Activity (U mg^−1^)	*K_m_* (µM)	*k_cat_* (s^−1^)	*k_cat_*/*K_m_* (µM^−1^ s^−1^)	TAI (Fold)
Parent-type	-	19.1 ± 1.1	205.7 ± 11.6	2208.2 ± 365.3	10.9 ± 2.4	1
IVA189	V160D, T292N, A491S	554.9 ± 41.9	69.8 ± 3.6	20,486.1 ± 569.2	293.9 ± 7.1	24.6 ± 2.5
IVA205	V160D, T290N, T292N, P303S, A491S	560.2 ± 28.4	85.3 ± 0.3	20,774.2 ± 827.1	243.5 ± 10.7	21.3 ± 0.8
IVA264	V160D, T290N, T292N, P303S, T412S	468.6 ± 137.0	64.0 ± 2.5	16,081.9 ± 5120.5	254.9 ± 90.0	26.5 ± 2.2

The results represent the mean ± standard deviation of duplicate independent experiments.

**Table 3:** Computed free energies of folding and energy components (Δ*G*) of the parent laccase and the relative free energies of folding (ΔΔ*G*) of the evolved laccases.

Energy Term	Δ*G* (kcal mol^−1^)	ΔΔ*G* (kcal mol^−1^)			Energy Description
	Parent-Type	IVA189	IVA205	IVA264	
Total Energy	200.01	−56.82	−37.53	−35.32	Overall stability of protein
Interaction Energy	2.73	−1.44	−0.23	−0.04	Interaction energy of subunits
Backbone H-bond	−602.47	−0.51	2.27	3.60	Contribution of backbone H-bonds
Sidechain H-bond	−301.57	5.45	10.44	12.77	Contribution of sidechain-sidechain and sidechain-backbone H-bonds
Van der Waals	−1196.69	1.95	3.59	3.76	Contribution of Van der Waals forces
Electrostatics	−53.25	−3.98	−6.34	−5.43	Electrostatic interactions
Solvation Polar	1619.42	1.15	7.02	5.18	Penalization for burying polar groups
Solvation Hydrophobic	−1557.13	2.99	7.29	7.10	Contribution of hydrophobic groups
Van der Waals clashes	168.32	−46.35	−39.64	−40.76	Energy penalization due to Van der Waals clashes (inter-residue)
Entropy Side Chain	535.27	0.77	1.82	0.75	Entropy cost of fixing the side chain
Entropy Main Chain	1530.27	2.37	−5.07	−4.38	Entropy cost of fixing the main chain
Cis Bond	9.94	−0.48	−0.73	−0.74	Cost of having a cis peptide bond
Torsional Clash	61.52	−20.18	−18.36	−17.36	Van der Waals torsional clashes (intra-residue)
Helix Dipole	−1.81	−0.78	−0.83	−0.86	Electrostatic contribution of the helix dipole
Disulfide	−20.12	0.05	0.06	0.06	Contribution of disulfide bonds
Electrostatic Kon	0.01	−0.05	−0.05	−0.04	Electrostatic interaction between molecules in the precomplex
Energy Ionization	8.30	0.76	1.00	1.03	Contribution of ionization energy

**Table 4:** Difference in pKa values (ΔpKa) between the parent laccase and evolved laccases.

Residue Name	ΔpKa		
	IVA189	IVA205	IVA264
V160D	4.18	4.17	4.17
V655D	4.09	4.04	4.04
Titratable group ^a^	9.87	11.82	12.30

^a^ Titratable group includes aspartic acid, arginine, glutamic acid, histidine and lysine.

## Supplementary Materials

The following are available online at http://www.mdpi.com/1422-0067/19/10/2989/s1.
