# Supplementary material for: Directed Evolution of a Homodimeric Laccase from Cerrena unicolor BBP6 by Random Mutagenesis and In Vivo Assembly
Source: Int J Mol Sci. 2018 Sep 30;19(10):2989. doi: 10.3390/ijms19102989 (PMC6213006; doi:10.3390/ijms19102989)
Supplement: Supplementary file 1 [file ijms-19-02989-s001.pdf]

Table S1. Comparison of laccases evolution.

Species	Methods of Evolution	Screened Clones	Rounds	Fold Improvement [a]						Evolved Properties	Ref.
				Substrates	K_m	k_{cat}	k_{cat}/K_m	Specific Activity	Total Activity		
Basidiomycete PM1	EpPCR, in *vivo* DNA shuffling, IvAM [b] and IVOE [c]	~50,300	8	ABTS, pH 5	ND [d]	ND	ND	ND	34,000	Improved laccase activity and thermostability	[22]
	EpPCR, in *vivo* DNA shuffling, IvAM, StEP,[e] Site-Directed and Saturation Mutagenesis	~5,100	4	ABTS, blood buffer pH 7.4	ND	0 to 143	ND	ND	41,840	Shifted pH profile and reduced chloride inhibition	[15]
T. versicolor	EpPCR	2800	2	ABTS, pH 4.5	0.9	3.3	2.9	ND	3.5	Improved laccase activity in ionic liquid	[16]
Myceliophthora thermophila	EpPCR, in *vivo* DNA shuffling, IvAM, StEP and Saturation Mutagenesis	>12000	5	ABTS, pH 7	2.1	14.4	30.9	ND	ND	Broader pH profile	[9]
				DMP, pH 7	0.5	17.6	9.2	ND	ND		
Pycnoporus cinnabarinus	EpPCR, in *vivo* DNA shuffling and IVOE	~7,600	6	ABTS, pH 5	1.5	12.7	18.4	ND	8,000	Improved laccase activity and shifted pH profile	[24]
				Sinapic acid, pH 5	0.6	9.2	5.1	ND	ND		
				DMP, pH 5	0.1	12.1	1.6	ND	ND		
Pycnoporus cinnabarinus and *PM1 basidiomycete*	Computer-aided site directed mutagenesis and IVOE	ND	1	Aniline, pH 3	0.5	2.2	1.1	ND	ND	Improved turnover rate	[12]
	ISM	>15000	1	Sinapic acid, pH 5	0.7	1.6	1.2	ND	ND	Shifted pH profile and enhanced turnover rate	[13]
Botrytis aclada	EpPCR, site-saturation	ND	4	ABTS, pH 3 to 6	up to 1.6	up to 1.8	up to 1.8	up to 4.8	ND	Improved laccase activity	[17]

	mutagenesis, site-directed mutagenesis			DMP, pH 3 to 6	up to 4.6	up to 1.8	up to 4.8	up to 2.1	ND	at pH 3-7.5 and thermostability	
Cerrena unicolor BBP6	EpPCR and *in vivo* assembly	~3,500	2	ABTS, pH 4	2.9	9.3	27.0	29.1	37.2	Improved laccase activity and thermostability, broader pH profile	This study

[a] All improvement data listed are from the best variant only.

[b] *In vivo* assembly of mutant libraries constructed with different mutational spectra.

[c] *In vivo* Overlap Extension.

[d] Not determined.

[e] *In vitro* recombination through staggered extension process.

Table S2. Primers used in the study.

Primers	Sequence (5' to 3')	Remarks
OL_pYEα-F	CCGAGCTCGGATCCACTAGTAACGGCCGCCAGTGTGCTGGAATTATGAGATTTCCTTCAATTTTTACTG	epPCR
OL_lac-R	GTGAATGTAAGCGTGACATAACTAATTACATGATGCGGCCCTCTAGATGCATGCTCGAGCGGCCGCTTACTTGTCGCCATCAGCAA	
αF_1F	TAGGGAATATTAAGCTTGGTACCGAGCTCGGATCCACTAGTAACGGCCGCCAGTGTGCTGGAATTCATGAGATTTCCTTCAATTWTTACT	Amplification of fragment αF1
αF_1R	TAATGCGGAGGATGCTGCGAATAAAACAGCAGTAAWAATTGAAGGAAATCTCATGAATTCCAGCACACTGGCGGCCGTTACTAGTGGATC	
αF_2F	CTGTTTTATTCGCAGCATCCTCCGCATTAGCTGCTCCAGTCAWCACTACAACA	Amplification of fragment αF2
αF_2R	CACAATGTGAATGTCGGTGACAGGACCAACGGCTCTTTTCTCGAGAGATACCCCTTCTTCTTTAGYAGCAATGCTG	
Lac_1F	ACTATTGCCAGCATTGCTGCTAAAGAAGAAGGGGTATCTCTCGAGAAAAGAGCCGTTGGT	Amplification of fragment Lac1
Lac_1R	AAGACCATTGATCAAGGTGGTATCAGCGATGGCAACACCGWCGATTTGASGGGCCAAAGTATGATACCAGTCGGCCAAAG	
Lac_2F	ATCGCTGATACCACCTTGATCAATGGTCTT	Amplification of fragment Lac2
Lac_2R	ACGGGGGTAGTGACCAGGGGATGGAGGTTGGRCTCCTGGAGAGGCTTGGTAGAAGTGGTCTGAKTGGTAKTAGGCTCAGCTACCGGTGCGCCTTTGTAGC	
Lac_3F	CAACCTCCATCCCCTGGTCACTACCCCCGT	Amplification of fragment Lac3
Lac_3R	GACAACATCACGAACGATAGGGTCAACGTAGTTGGGASTAGTTTGACCGGCACTGCGAACAAC	
Lac_4F	CTACGTTGACCCTATCGTTCGTGATGTTGTC	Amplification of fragment Lac4
Lac_4R	TGAATGTAAGCGTGACATAACTAATTACATGATGCGGCCCTCTAGATGCATGCTCGGCGGCCGCTTACTTGTCGCCATCAGMAAGAGCAT	
T7_promoter	TAATACGACTCACTATAGGG	DNA sequencing
pYEsqR	CGGTTAGAGCGGATGTGGG	
αF54_F	TAGGGAATATTAAGCTTGGTACCGAGCTCGGATCCACTAGTAACGGCCGCCAGTGTGCTGGAATTCATGAGATTTCCTTCAA	Amplification of evolved α-factor
αF54_R	CACAATGTGAATGTCGGTGACAGGACCAACGGCTCTTTTCTCGAGAGATACCCCTTCTTCTTTAG	
Lac_uni_F	CTAAAGAAGAAGGGGTATCTCTCGAGAAAAGAGCCGTTGGTCCTGTCACCGACATTCACATTGTG	Amplification of evolved laccases
Lac_uni_R	TGAATGTAAGCGTGACATAACTAATTACATGATGCGGCCCTCTAGATGCATGCTCGGCGGCCGCTTACTTGTCGCCATC	
